# Pet Rats as the Likely Reservoir for Human Seoul Orthohantavirus Infection

**DOI:** 10.3390/v15020467

**Published:** 2023-02-07

**Authors:** Elisa Heuser, Stephan Drewes, Jakob Trimpert, Dusan Kunec, Calvin Mehl, Marieke P. de Cock, Ankje de Vries, Christiane Klier, Martin Oskamp, Peter Tenhaken, Fatima Hashemi, Daniela Heinz, Mariana Nascimento, Marc Boelhauve, Rasa Petraityte-Burneikiene, Dina Raafat, Miriam Maas, Detlev H. Krüger, Andreas Latz, Jörg Hofmann, Gerald Heckel, Johannes Dreesman, Rainer G. Ulrich

**Affiliations:** 1 Institute of Novel and Emerging Infectious Diseases, Friedrich-Loeffler-Institut, Federal Research Institute for Animal Health, 17493 Greifswald, Germany; elisa.heuser@fli.de (E.H.); stephan.drewes@fli.de (S.D.); calvin.mehl@fli.de (C.M.); 2 German Center for Infection Research (DZIF), Partner Site Hamburg-Lübeck-Borstel-Riems, 17493 Greifswald, Germany; 3 Institute of Virology, Department of Veterinary Medicine, Freie Universität Berlin, 14163 Berlin, Germany; jakob.trimpert@fu-berlin.de (J.T.); dusan.kunec@fu-berlin.de (D.K.); m.nascimento@fu-berlin.de (M.N.); 4 Centre for Infectious Disease Control, National Institute for Public Health and the Environment (RIVM), 3720 BA Bilthoven, The Netherlands; marieke.de.cock@rivm.nl (M.P.d.C.); ankje.de.vries@rivm.nl (A.d.V.); miriam.maas@rivm.nl (M.M.); 5 Department of Infectious Diseases, Public Health Agency of Lower Saxony, 30449 Hannover, Germany; johannes.dreesman@nlga.niedersachsen.de (J.D.); christiane.klier@nlga.niedersachsen.de (C.K.); 6 Health Department of Grafschaft Bentheim County, 48527 Nordhorn, Germany; martin.oskamp@grafschaft.de; 7 Health service for the district and city of Osnabrück, 49082 Osnabrück, Germany; peter.tenhaken@lkos.de; 8 Veterinary Department, NovaTec Immundiagnostica GmbH, 64859 Dietzenbach, Germany; fatimahashemi95@gmail.com (F.H.); andreas.latz@goldstandarddiagnostics.eu (A.L.); daniela.heinz@goldstandarddiagnostics.eu (D.H.); 9 University of Applied Sciences, 60318 Frankfurt am Main, Germany; 10 Department of Agriculture, South Westphalia University of Applied Sciences, 59494 Soest, Germany; boelhauve.marc@fh-swf.de; 11 Institute of Biotechnology, Life Sciences Center, Vilnius University, LT-10257 Vilnius, Lithuania; rasa.burneikiene@bti.vu.lt; 12 Institute of Immunology, University Medicine Greifswald, 17475 Greifswald, Germany; dina.raafat@med.uni-greifswald.de; 13 Department of Microbiology and Immunology, Faculty of Pharmacy, Alexandria University, Alexandria 21521, Egypt; 14 Institute of Virology, Charité–University Medicine Berlin, 10117 Berlin, Germany; joerg.hofmann@charite.de (J.H.); detlev.krueger@charite.de (D.H.K.); 15 Institute of Ecology and Evolution, University of Bern, 3012 Bern, Switzerland; gerald.heckel@iee.unibe.ch

**Keywords:** Seoul virus, pet rat, Norway rat, high-throughput sequencing, complete coding sequences, rat surveillance, zoonoses

## Abstract

Seoul orthohantavirus (SEOV) is a rat-associated zoonotic pathogen with an almost worldwide distribution. In 2019, the first autochthonous human case of SEOV-induced hemorrhagic fever with renal syndrome was reported in Germany, and a pet rat was identified as the source of the zoonotic infection. To further investigate the SEOV reservoir, additional rats from the patient and another owner, all of which were purchased from the same vendor, were tested. SEOV RNA and anti-SEOV antibodies were found in both of the patient’s rats and in two of the three rats belonging to the other owner. The complete coding sequences of the small (S), medium (M), and large (L) segments obtained from one rat per owner exhibited a high sequence similarity to SEOV strains of breeder rat or human origin from the Netherlands, France, the USA, and Great Britain. Serological screening of 490 rats from breeding facilities and 563 wild rats from Germany (2007–2020) as well as 594 wild rats from the Netherlands (2013–2021) revealed 1 and 6 seropositive individuals, respectively. However, SEOV RNA was not detected in any of these animals. Increased surveillance of pet, breeder, and wild rats is needed to identify the origin of the SEOV strain in Europe and to develop measures to prevent transmission to the human population.

## 1. Introduction

Hantaviruses are (re-)emerging zoonotic pathogens that are transmitted by small mammals, especially rats, voles, and mice [1]. Their genome consists of three RNA segments of negative polarity: the small (S) and medium (M) segments encode the structural proteins of the virion, the nucleocapsid (N) protein, and the glycoprotein precursor, respectively. The large (L) segment encodes the RNA-directed RNA polymerase [2]. In humans, hantavirus infection can cause hemorrhagic fever with renal syndrome (HFRS) and hantavirus cardiopulmonary syndrome. Seoul orthohantavirus (SEOV) causes a mild to moderate form of HFRS with initial symptoms such as fever, chills, headache, vomiting, and rash. The case fatality rate was estimated at about 1% [3,4]. Hantavirus isolation or viral RNA recovery from human samples are uncommon because the virus is often cleared in the organism before patient samples are collected for diagnostic approaches [5].

SEOV was first discovered in a Norway (or brown) rat (*Rattus norvegicus*) captured in Seoul, South Korea, in 1980 [6]. The virus was later detected in other parts of Asia, although with a lower prevalence than the Hantaan orthohantavirus (HTNV) [7,8]. Further investigations in Asia detected SEOV not only in Norway rats but also in black (or roof) rats (*Rattus rattus*) and other *Rattus* species [8]. Since then, SEOV has been detected on almost all continents following the global distribution of these synanthropic rodents [9,10,11,12].

In Europe, several studies evaluated the occurrence of SEOV in pet, breeder, and wild rats and detected human disease cases caused by SEOV transmission by pet rats [13,14]. SEOV, which was detected in pet and breeder rats from Great Britain [15] and the Netherlands [16] as well as in a pet rat in Sweden that had been imported from England [17], was associated with human disease cases [13,14]. Studies on wild rats from Belgium indicated the presence of SEOV-reactive antibodies [18]. Furthermore, studies in the Eastern province of Gelderland in the Netherlands and in pig farms in Great Britain in 2013 showed SEOV RNA in wild rats [19,20]. In Germany, a single human SEOV infection was reported in 2018 in a traveler returning from Southeast Asia [7], while other studies using human serum panels from Germany failed to detect SEOV-reactive antibodies using neutralization assays [21,22].

In October 2019, a female patient with a high fever who was admitted to the intensive care unit of a hospital in Lower Saxony (Germany) in critical condition was diagnosed with SEOV infection via conventional reverse transcription-polymerase chain reaction (RT-PCR). Her pet rat was presumed to be the source of infection [23]. An analysis of partial SEOV sequences from the patient and her pet rat indicated a large similarity to pet-and breeder-rat-derived sequences from the Netherlands, France, Great Britain, and the USA, but they differed from sequences found in wild rats from the Netherlands, Great Britain, and other countries. At that time, the occurrence of SEOV in breeder and wild rats in Germany was not investigated.

The two main objectives of this study were: (i) to identify more precisely the origin of the human SEOV infection in Germany based on epidemiological trace-back and trace-forward investigations; and (ii) to evaluate the potential origin of the SEOV strain in the patient by investigating pet, breeder, and wild rats from both Germany and the Netherlands.

## 2. Materials and Methods

### 2.1. Epidemiological Investigation of the Origin of the Human SEOV Infection in Germany

Once a pet rat was identified as the likely source of the human SEOV infection in 2019 [23], the origin of the pet rats was followed by tracing the distribution channels of the pet rats back and forth (Supplementary Figure S1). Local public health and veterinary authorities as well as members of the research team methodologically conducted interviews and home visits with the patient (pet-rat owner 1), the vendor, and another pet-rat owner (pet-rat owner 2) who purchased the rats from the same vendor.

### 2.2. Collection of Rats

**Figure 1:**
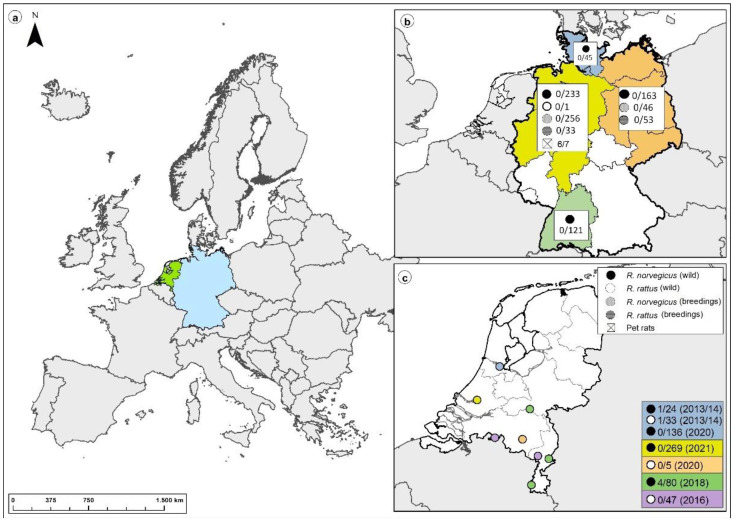
Location of Norway and black rat collection sites in Europe (**a**), Germany (wild-trapped rats and rats from breeding colonies) (**b**), and in the Netherlands (wild-trapped rats) (**c**). Map of the Netherlands (**c**): Amsterdam (blue); Rotterdam (yellow); Eindhoven (orange); Appeltern, Roermond, and Maastricht (green); and various municipalities of North Brabant and Limburg (purple).

In addition to the patient’s first pet rat (KS19/1354) [23], three more pet rats from pet-rat owner 1 and three from pet-rat owner 2 were collected, euthanized, and sent to the National Reference Laboratory for Hantaviruses at the Friedrich-Loeffler-Institut (FLI), Greifswald-Insel Riems, Germany. In addition, we included materials from earlier studies comprising samples of rats from breeding facilities and wild populations across Germany (2007 to 2019 [24,25]), from wild populations from rural and urban areas in the Netherlands (2013–2021), as well as from ongoing studies of commensal rodent pathogens in Germany (2020; see Figure 1 and Supplementary Table S1). The rats from breeding facilities in Germany comprised laboratory Norway rats, wild-trapped Norway and black rats, as well as feeder rats from a zoo. Rats captured in 2013–2014 in the Netherlands were live-trapped with methods described previously [26]; other rats were captured using snap-traps. All carcasses were stored frozen at −20 °C until dissection.

### 2.3. Dissection of Rats

Following standard protocols, the frozen rat carcasses were thawed at 4 °C and dissected. A standardized panel of organs (lung, heart, liver, spleen, blood/transudate, and kidney) was taken from rats in Germany for use in the screening for SEOV and stored at −20 °C until further investigation. In the Netherlands, lung samples were collected and stored in RNAlater^®^ (Thermo Fisher Scientific, Landsmeer, The Netherlands) for 3 to 5 days before storage at −80 °C until they were further processed.

### 2.4. Rat Control Sera

SEOV-positive control sera (L and R) were generated via four-time immunization of two Norway rats in four-week intervals with 10 µg of yeast-expressed, purified, and His-tagged SEOV N protein [27]. For immunization, the antigen was mixed 1:1 with 100 µL of Adjuvans Gerbu MM (Gerbu Biotechnik GmbH, Heidelberg, Germany) and injected subcutaneously. Serum and thoracic lavage from a non-infected Norway rat from the breeding facility at FLI were used as a negative control.

### 2.5. Screening Strategy for the Different Rat Panels

As this study combined results from rats belonging to four different panels (I–IV) that were collected over a long period of time (15 years), slightly different procedures were used over the years (see Supplementary Table S1 and below). The pet rats from Germany (panel I) were investigated using a SEOV-immunoglobulin G (IgG) enzyme-linked immunosorbent assay (ELISA) (A), a hantavirus IgG ELISA (B), a line-blot assay, and conventional RT-PCR assay. The other rats from Germany (panels II and III) were investigated using an IgG ELISA (B), a line-blot assay, and RT-PCR. Rats collected in the Netherlands (panel IV) were all tested using an SEOV-IgG ELISA (C) and/or real-time RT-PCR [28]. All seropositive rat samples from the Netherlands (collected during 2016–2021) were also investigated via a virus neutralization test that used SEOV, Puumala orthohantavirus (PUUV), Dobrava-Belgrade orthohantavirus (DOBV), and Tula orthohantavirus (TULV) as previously described [29].

### 2.6. ELISA

#### 2.6.1. SEOV-IgG ELISA (A)

For the samples collected from German pet rats (panel I), a SEOV-IgG ELISA was performed using thoracic lavage (diluted 1:10) and whole blood (diluted 1:200) of the rats. A yeast-expressed, His-tagged, and affinity-purified SEOV N-protein served as the antigen [27]. A microtiter plate (C96 PolySorp™, NUNC, VWR, Darmstadt, Germany) was coated with 100 µg/well of the antigen in carbonate buffer and incubated overnight at 4 °C. Thereafter, the plate was blocked with 1% bovine serum albumin (BSA; Serva, Heidelberg, Germany) in phosphate-buffered saline (PBS)/0.05% Tween20 (Merck, Darmstadt, Germany) for 1 h at room temperature. The plate was then inoculated with the samples (diluted with 0.5% BSA in PBS/0.05% Tween20) and incubated for 1 h at 37 °C, which was followed by a wash step with washing buffer (PBS/0.1% Tween20), then the addition of horseradish peroxidase (HRP)-labeled anti-rat IgG conjugate (1:12,500 100 µL) and a further 1 h incubation at 37 °C. The plate was washed again and then incubated with 100 µL of 3,3′,5,5′-tetramethylbenzidine (TMB) (BioRad, Munich, Germany) for 10 min, and the reaction was then stopped by adding 100 µL of 1 M sulfuric acid. Finally, the plate was measured in an ELISA plate reader Infinite® M200 Pro (Tecan, Männedorf, Switzerland) at 450 nm. The upper and lower cut-off values were defined as previously described [30]. Samples with an optical density (OD) value below the lower cut-off value were defined as negative and those with OD values above the upper cut-off value as positive. OD values lying between the upper and lower cut-off values were considered to be inconclusive. Positive and inconclusive samples were retested according to a previously published decision tree [30].

#### 2.6.2. Hantavirus-IgG ELISA (B) 

For the hantavirus IgG ELISA (NovaTec Immundiagnostica GmbH, Dietzenbach, Germany) that targeted multiple hantaviruses, medium-binding 96-well ELISA plates (Greiner, Nürtingen, Germany) were coated with a mixture of four antigens (DOBV, PUUV, HTNV, and SEOV) using 100 ng/well of each individual antigen in carbonate-buffer (NovaTec Immundiagnostica GmbH) and left overnight at 4 °C. The plates were washed once with 250 µL wash-after-coat buffer (NovaTec Immundiagnostica GmbH) and blocked with stabilization and blocking buffer (NovaTec Immundiagnostica GmbH) for 1 h at room temperature. After drying for 30 min at 37 °C, the plates were stored at 4 °C until further use. Thoracic lavage or blood samples of rat panels I-III were diluted 1:100 in sample dilution buffer (NovaTec Immundiagnostica GmbH). Aliquots of diluted samples (100 µL/well) were incubated for 1 h at 37 °C followed by 3 washes with washing buffer (0.2 M phosphate, pH = 7.2; NovaTec Immundiagnostica GmbH) and 30 min of incubation at room temperature with 100 µL/well of a titrated dilution of a HRP-labeled protein A/G conjugate (NovaTec Immundiagnostica GmbH; final dilution 1:900,000 of a 5 mg/mL stock solution). After three more washes with washing buffer, the plates were incubated for 15 min at room temperature with TMB substrate solution (NovaTec Immundiagnostica GmbH). The reaction was stopped by adding 100 µL/well of stop solution (0.2 M sulfuric acid; NovaTec Immundiagnostica GmbH) and evaluated by measuring at 450 nm in a plate reader (Anthos Labtec Instruments, Wals-Siezenheim, Austria).

#### 2.6.3. SEOV-IgG ELISA (C)

Sera taken from rats from the Netherlands (panel IV) were tested for the presence of anti-SEOV IgG antibodies using a commercial human DOBV/HTNV IgG ELISA (Progen Biotechnik GmbH, Heidelberg, Germany), that was adapted to detect rat IgG antibodies by using rabbit-anti-rat HRP-labeled IgG conjugate (Sigma–Aldrich Chemie B.V. Zwijndrecht, The Netherlands) at a 1:5000 dilution. The optimal OD cut-off value was determined through the use of a binary mixture model as previously described [26].

### 2.7. Line-Blot Analysis

For the line-blot format, the recombinant antigens of DOBV, PUUV, HTNV, SEOV, Andes orthohantavirus, and Sin Nombre orthohantavirus were printed with a dispenser (FrontLine HR microliter contact; BioDot, Irvine, CA, USA) on a nitrocellulose membrane (GE Healthcare, Chicago, IL, USA) together with a control line for sample loading and conjugate function. After drying, the membranes were cut into 3 mm strips and stored at 4 °C. Prior to use, strips were equilibrated in 1 mL of sample dilution buffer (10 mM phosphate buffer, pH 7.2; NovaTec Immundiagnostica GmbH). Rat samples (panels I–III) were added to the membranes, and then the membranes were incubated with gentle shaking for 1 h at room temperature. After 3 washes with 1 mL of washing buffer (0.2 M phosphate, pH = 7.2; NovaTec Immundiagnostica GmbH) for 5 min each, the membranes were incubated with gentle shaking for 30 min with 1 mL of HRP-labeled protein A/G conjugate (NovaTec Immundiagnostica GmbH) at room temperature. The strips were then washed three times with 1 mL of washing buffer for 5 min. Signals were developed by incubating the membranes in 1 mL of TMB substrate solution (NovaTec Immundiagnostica GmbH) for 15 min with gentle shaking at room temperature. The reaction was stopped by the addition of at least 1 mL of distilled water. After drying the membranes for at least 30 min at room temperature, the strips were visually evaluated.

### 2.8. SEOV RNA Detection and Sequence Determination

For SEOV RNA detection and tissue-distribution analysis in rats from Germany, nucleic acids were extracted manually from homogenized heart, lung, liver, kidney, and spleen tissues using QIAzol Lysis Reagent (Qiagen, Hilden, Germany) or a NucleoMag^®^ VET kit (Macherey-Nagel, Düren, Germany) according to the manufacturer’s instructions on a KingFisher™ Flex Purification System (Thermo Fisher Scientific, Waltham, MA, USA). The pet rats of the patient/pet-rat owner 1 were initially investigated using a hantavirus L segment-specific conventional one-step RT-PCR using primers Han-LF1: ATG TAY GTB AGT GCW GAT GC and Han-LR1: AAC CAD TCW GTY CCR TCA TC [31] and thereafter with conventional S segment-RT-PCR using the following primers: SEOV_415_alt_fw: TAC ATG TTA ACA ACA AGA GG and SEOV_1130_alt_rev: TCC AGT TGT ATT CCC ATT GAT TG for the S segment. These novel primers were designed based on an alignment of representative SEOV S segment sequences from Europe that included the pet-rat-derived sequence (KS19/1354). The three RT-PCR positive rats from pet-rat owner 1 were also investigated for the M segment using primers SEOV_M_47_fw: CTC TGG ACA GAT AAT GCT CAT G and SEOV_M_1068_rev: CCC AGC TAT TTT CAT ACT CAT AAT C. The rats from pet-rat owner 2 were investigated using the conventional S segment RT-PCR alone. The one-step RT-PCRs were performed using the SuperScript III RT-PCR Kit (Qiagen). Breeder and wild rats from Germany (panels II and III) were investigated using conventional S segment RT-PCR (above). RT-PCR products were resolved via electrophoresis in a 1.5% agarose gel with subsequent Safe View (Applied Biological Materials, Richmond, BC, Canada) or ethidium bromide (Sigma-Aldrich, St. Louis, MO, USA) staining. A BigDye™ Terminator v1.1 Cycle Sequencing Kit (Applied Biosystems, Waltham, MA, USA) was used for Sanger sequencing. Consensus sequences were created using BioEdit v7.2.5 [32].

For the rats from the Netherlands (2013/2014, subset of panel IV), the lung tissue was manually disrupted in liquid nitrogen and RNA was extracted using a RNeasy isolation minikit (Qiagen) and including a Qiashredder step for homogenization and DNase I treatment. From 2016 onward, lung tissue samples were homogenized two times (40 sec at maximum speed) using MagNA Lyser Green Bead tube (Roche Diagnostics, GmbH, Mannheim, Germany) with 500 µL of lysis buffer (MagNa Pure 96 Total Nucleic Acid Isolation kit) on a FastPrep FP120 homogenizer (Thermo Fisher Scientific). Total nucleic acid was extracted using a MagNa Pure 96 Total Nucleic Acid Isolation kit on a MagNA pure 96 (Roche Diagnostics). Quality control of the RNA isolation and inhibition control was performed using β-actin RT-PCR. Samples were subsequently tested via real-time RT-PCR that targeted the S segment [28]. From 2013 to 2018, TaqMan Master (Roche Diagnostics) was used, while in subsequent years TaqMan Fast Virus 1-Step Master Mix was used (Thermo Fisher Scientific). The black rats that were captured in 2013–2014 were also tested using a nested conventional RT-PCR assay of the L segment as described previously [31].

### 2.9. Enrichment of Target Viral Sequences and High-Throughput Sequencing (HTS)

For the HTS libraries, 1 µg of total spleen and liver RNA from one rat from each pet-rat owner was processed using a NEBNext Ultra II RNA library preparation kit (New England Biolabs, Ipswich, MA, USA) according to the manufacturer’s instructions with eight minutes of RNA fragmentation and seven PCR cycles to amplify the resulting cDNA libraries. Amplified cDNA libraries were enriched for hantavirus sequences using a custom-made myBaits tiling array (Arbor Biosiences, Ann Arbor, MI, USA) designed to capture sequences from rodent-associated orthohantaviruses according to the manufacturer’s instructions. Briefly, 1 µg of HTS library was hybridized for 20 hours to biotinylated RNA baits, captured with streptavidin-coated paramagnetic beads, washed extensively to remove non-target sequences, and eluted from the beads through heat denaturation. Enriched sequences were amplified with 10 cycles of PCR using Q5 high-fidelity DNA polymerase (New England Biolabs) and primers annealed to the P5 and P7 library sequences. Enriched and amplified libraries were run on an Illumina MiSeq sequencer using v.3 chemistry for 2 × 300 bp paired-end reads.

Raw sequencing data were processed using Trimmomatic v.0.39 [33] and mapped against the genome of SEOV strain 201701093/SEOV/Illinois_US/Rat (GenBank accession: MK360784 (S segment), MK360797 (M segment), and MK360803 (L segment)) using the Burrows-Wheeler aligner v0.7.17 [34]. Mapping statistics were generated using Samtools v.1.10 [35], and alignments were visualized using IGV v2.9.4 for Linux [36]. For detection of single-nucleotide polymorphisms (SNPs), Freebayes (a Bayesian genetic variant detector) was used [37]. All SNPs with a minimum mapping quality of 5, a minimum count of 3, and a minimum fraction of 0.1 were considered. Consensus sequences for each sample were obtained using BCFtools [35].

### 2.10. Sequence Comparison and Phylogenetic Analyses

Sequences were identified using Basic Local Alignment Search Tool (BLAST, National Center for Biotechnology Information, NCBI) [38] and aligned using BioEdit v7.2.5 [32]. The best-fitting substitution model was determined using jModeltest 2.1.10 [39]. Phylogenetic tree reconstruction was performed using the CIPRES Science Gateway v3.3 with the implemented programs FastTreeMP on XSEDE v2.1.10 for maximum likelihood tree reconstruction and MrBayes on XSEDE v3.2.7a for Bayesian tree inference [40,41,42]. Subsequently, consensus trees were constructed based on Bayesian trees. For this purpose, bootstrap values were transferred from the maximum likelihood tree to the consensus tree if branches were congruent.

## 3. Results

### 3.1. Identification of the Origin of the Human SEOV Infection

The epidemiological investigation identified pet rats as a possible source of the laboratory-confirmed human SEOV infection in a patient (pet-rat owner 1) in Lower Saxony (Germany) [23]. A trace-back investigation revealed that the patient had purchased the infected index pet rat (KS19/1354) and two additional juvenile rats from a vendor in North Rhine-Westphalia (Germany) via an internet marketplace approximately three months before the onset of symptoms. The investigation also revealed that the patient already had owned an older female pet rat for some time before the onset of the disease that was kept separate from the new male rats (Supplementary Figure S1). After the first rat [23] and two additional rats (see below) tested positive, the patient agreed to euthanize and test the older fourth rat.

A trace-forward investigation at the vendor’s residence revealed that three other rats had been sold to another private customer (pet-rat owner 2) in Lower Saxony (Germany; Supplementary Figure S1). The responsible local health department contacted and informed pet-rat owner 2 about the potential risk of keeping pet rats that were potentially infected with SEOV. Pet-rat owner 2, who reported not having any signs of illness, agreed to sacrifice the rats for further investigations at the FLI, Germany, in December 2019.

The vendor himself had previously bought eight rats at a reptile market in North Rhine-Westphalia, Germany. There, the rats had been sold outside of the official market as feeder rats. No further trace-back was possible on the origin of the rats sold at the reptile market.

### 3.2. Analysis of Rats from Patient/Pet-Rat Owner 1 and Pet-Rat Owner 2

The analysis of the partial sequence of one of the patient’s pet rats (KS19/1354) revealed the potential origin of the patient’s infection due to a nucleotide sequence identity of 99.8% for the S segment and a 100% identity for the L segment (data not shown [23]). The patient’s other two juvenile rats (KS19/1352 and KS19/1353) also tested positive in the conventional S and M segment RT-PCRs, but only one (KS19/1352) was positive in the conventional L segment RT-PCR. The patient’s fourth, older rat (KS19/1414), which was obtained from a different source, tested negative in the S segment RT-PCR assay (Table 1).

The parallel analyses of five different tissues of the RT-PCR-positive pet rats revealed two to five RT-PCR-positive tissues (Supplementary Table S2). The SEOV-IgG ELISA (A) and hantavirus IgG ELISA (B) detected SEOV-reactive antibodies in all three rats of pet-rat owner 1 that originated from a vendor in North Rhine-Westphalia (Germany), but no antibodies were detected in the older rat (KS19/1414) obtained from a different source (Table 1). These results were confirmed by a line-blot assay (Supplementary Figure S2).

An analysis of the three rats from pet-rat owner 2 revealed that two of them tested positive for SEOV-reactive antibodies and SEOV RNA (Table 1 and Supplementary Figure S2).

**Figure 2:**
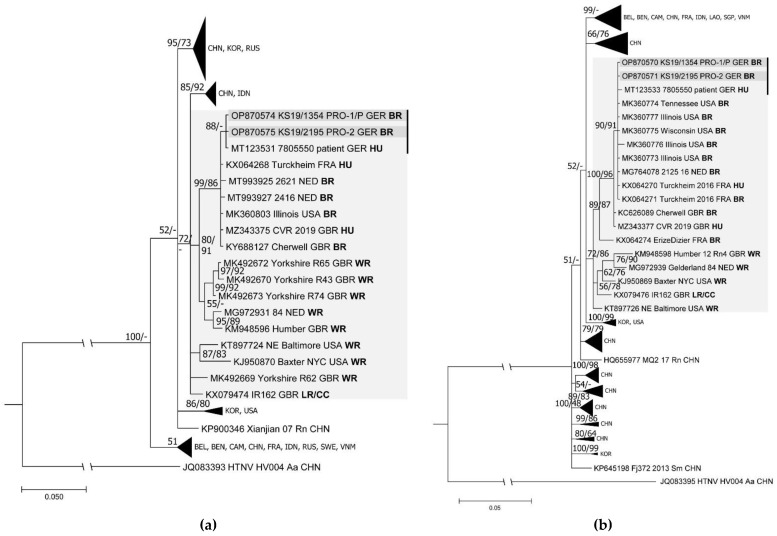
Phylogenetic trees of partial Seoul virus (SEOV) sequences from patient and rats. (**a**) The partial L segment consensus tree was based on the HKY substitution model with gamma distribution for Bayesian analyses of 8 × 10^6^ generations and the TPM1uf substitution model with gamma distribution and invariant sites for maximum likelihood analyses with an alignment length of 291 nucleotides and 1000 bootstrap replicates. (**b**) The partial S segment consensus tree was reconstructed using the TPM2 substitution model with gamma distribution for both the Bayesian (1 × 10^7^ generations) and maximum likelihood analyses (1000 bootstrap replicates) using sequences of 515 nucleotides in length. The consensus trees were based on the Bayesian method. Bootstrap values from the maximum likelihood analyses were only transferred to the consensus tree if the branches were consistent. Posterior probabilities are given before and bootstrap values behind or below slashes. Relevant clades are highlighted by a light grey background. Sequences derived from the same outbreak are marked by a black bar, while novel sequences from the pet rats are indicated in darker grey. Country abbreviations: BEL, Belgium; BEN, Benin; CAM, Cambodia; CHN, China; FRA, France; GBR, Great Britain; GER, Germany; IDN, Indonesia; KOR, Korea; LAO, Laos; NED, the Netherlands; RUS, Russia; SGP, Singapore; SWE, Sweden; USA, United States of America; VNM, Vietnam. Other abbreviations: HU, human; PRO-1/P, pet-rat owner 1/patient; PRO-2, pet-rat owner 2; Rn, *Rattus norvegicus*; BR, breeder rat; CC, cell culture isolate; LR, laboratory rat; WR, wild rat. The categorization of the rats was according to [10,19,20,43,44,45,46,47].

A pairwise comparison of the partial S and L segment sequences revealed almost identical sequences for the SEOV-RNA-positive rats and the patient-derived sequences as well as a very high similarity to sequences from pet rats in the USA, Great Britain, France, and the Netherlands (Supplementary Table S3). The phylogenetic analysis of these partial L and S segment sequences confirmed the close phylogenetic relationship of all sequences from the German pet rats and the German patient with those from pet rats and patients in France, the Netherlands, Great Britain, and the USA (Figure 2a,b).

### 3.3. Complete Genome Identification and Phylogenetic Analysis

The complete coding sequences (CDSs) of two SEOV strains were determined via target enrichment-based HTS. The first strain originated from a pet rat that belonged to the patient/pet-rat owner 1 (KS19/1354; a partial sequence was already published [23]), and the second strain originated from a rat that belonged to pet-rat owner 2 (KS19/2195). The CDSs and amino acid (aa) sequences of both strains were almost identical for all three segments and the corresponding encoded proteins (Supplementary Table S4). The CDSs of the S, M, and L segments were 1290 nucleotides (nts), 3402 nts, and 6456 nts long (including the stop codon) and encoded proteins of 429 aa, 1133 aa, and 2151 aa residues, respectively.

**Figure 3:**
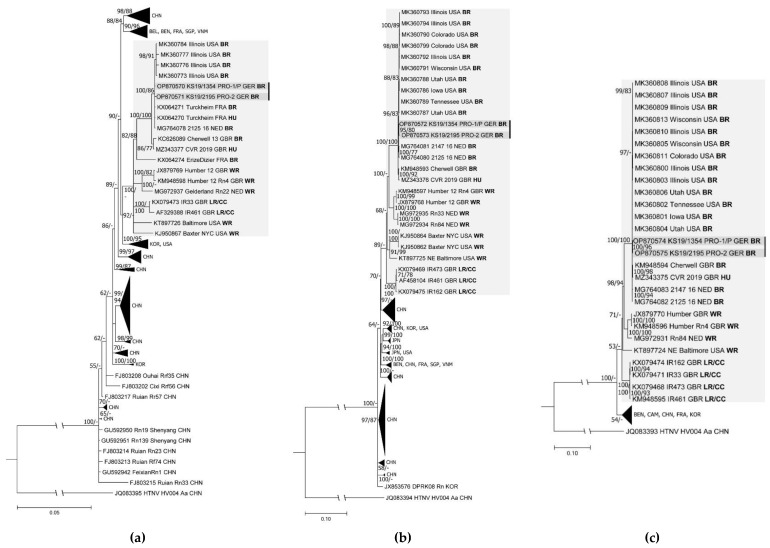
Phylogenetic trees of complete coding Seoul virus (SEOV) sequences of the S, M, and L segments. (**a**) The consensus tree of the complete coding S segment was derived from Bayesian analyses with 8 × 10^6^ generations using the TIM1 substitution model with gamma distribution and invariant sites. The maximum likelihood reconstructions were performed using the TrN substitution model with gamma distribution and invariant sites on a sequence length of 1290 nucleotides and 1000 bootstrap replicates. (**b**) The complete coding M segment consensus tree was reconstructed using Bayesian algorithms with 8 × 10^6^ generations and the TIM1 substitution model including gamma distribution and invariant sites. Maximum likelihood analyses used the GTR substitution model including gamma distribution and invariant sites based on a sequence length of 3402 nucleotides and 1000 bootstrap replicates. (**c**) The L segment complete coding consensus tree was reconstructed using the GTR substitution model with gamma distribution and invariant sites for Bayesian and maximum likelihood algorithms with 1 × 10^7^ generations and 1000 bootstrap replicates, respectively, with an alignment length of 6456 nucleotides. Bootstrap values were only transferred from the maximum likelihood trees to establish consensus trees if the branches were identical. Posterior probabilities are given before and bootstrap values behind or below slashes. Relevant clades are highlighted in a light grey background. Sequences derived from the same outbreak are marked by a black bar, while novel sequences from the pet rats are indicated in a darker grey background. Country abbreviations: BEL, Belgium; BEN, Benin; CAM, Cambodia; CHN, China; FRA, France; GBR, Great Britain; GER, Germany; JPN, Japan; KOR, Korea; NED, the Netherlands; SGP, Singapore; USA, United States of America; VNM, Vietnam. Other abbreviations: HU, human; PRO-1/P, pet-rat owner 1/patient; PRO-2, pet-rat owner 2; Rn, *Rattus norvegicus*; BR, breeder rat; CC, cell culture isolate; LR, laboratory rat; WR, wild rat. The categorization of the rats was according to [10,19,20,43,44,45,46,47].

A pairwise comparison of the S segment nt sequences of the rat of the patient/pet-rat owner 1 and the rat of pet-rat owner 2 confirmed their very high sequence similarity (99.7–100%) with sequences from breeder rats from the USA, Great Britain, France, and the Netherlands and from an SEOV patient in Great Britain (Supplementary Table S4). The S, M, and L segment sequences of both pet-rat-derived SEOV strains from Germany formed in the phylogenetic trees clusters with sequences from France, the Netherlands, Great Britain, and the USA (Figure 3a–c). These sequences were well separated in the phylogenetic trees from sequences from Belgium, China, Korea, and additional sequences from France and the USA. Similar results were obtained for partial M segment sequences of both pet rats deduced from the HTS dataset (Supplementary Figure S3).

### 3.4. Large-Scale Screening of Non-Pet Rats from Germany and The Netherlands

Six German scientific breeding facilities for Norway rats and black rats (panel II) were screened for SEOV RNA and SEOV-reactive antibodies. None of the investigated 404 Norway rats and 86 black rats showed any indication of an SEOV infection (Table 2).

In addition, 562 wild Norway rats and 1 wild black rat from urban and rural sites, livestock farms, and zoological gardens (pest rats) in Germany (panel III) were screened for SEOV RNA and SEOV-reactive antibodies. SEOV RNA was not detected in any of the investigated rats (Table 3). In the hantavirus IgG ELISA (B), a single rat from an agricultural farm in North Rhine-Westphalia tested positive, but only for the PUUV antigen in the line-blot assay (Table 3).

Of the 594 investigated rats from the Netherlands (panel IV), 6 tested positive in the IgG ELISA (C) (2013–2014: 1 *R. rattus* and 1 *R. norvegicus*; 2018: 4 *R. norvegicus*), all of which were negative in the real-time RT-PCR (Table 4). The four seropositive rats from 2018 were additionally tested via a virus neutralization assay using SEOV, DOBV, TULV, and PUUV that also turned out to be negative for SEOV and the other orthohantaviruses tested.

## 4. Discussion

This investigation of more than 1500 rats allowed for a broad overview of SEOV in pet, wild, and breeder rats in Germany and the Netherlands that was initiated after the first autochthonous human SEOV infection was discovered in Germany in 2019 [23]. In line with the detection of SEOV RNA in a patient’s rat [23], SEOV RNA was detected in two other pet rats of the same patient. The partial S and L segment SEOV sequences from the patient were almost identical to the novel rat-derived sequences detected here. In addition, all three pet rats tested positive for SEOV-reactive antibodies. The negative results in both SEOV RT-PCR and serology for a single older (female) pet rat in the household of the patient/pet-rat owner 1 suggested that the patient’s illness was caused by the recently purchased juvenile male rats. It might also indicate that the positive pet rats became infected at the breeding facility, whereas the patient’s older animal was refractory to infection due to the separate housing of male and female rats at the patient´s residence and possibly due to the lower infection rate in females [48].

Tracing back and forward activities resulted in the detection of additional SEOV-infected rats from pet-rat owner 2. The nucleotide sequences of strains from both the patient/pet-rat owner 1 and pet-rat owner 2 rats were almost identical, which suggested a common source of the virus strain. A phylogenetic analysis of the sequences revealed (nearly) identical SEOV sequences in breeder rats from France, the Netherlands and USA. This level of similarity was in contrast to previous findings concerning other human pathogenic hantaviruses; e.g., PUUV, for which a clear geographic clustering of sequences was observed that indicate virus evolution in isolated reservoirs of bank vole populations [49].

The high sequence similarity of the European pet and breeder rat SEOV sequences (even to sequences from breeder rats in the USA) suggested that there might be intensive private exchange of pet rats between neighboring European countries and even between Europe and the USA that is bypassing official trade channels. The trade of pet rats partly overlaps with the trade of feeder rats, as the history of the rats from pet-rat owners 1 and 2 showed, and was also reported previously [50]. However, the current study found no evidence of SEOV in breeder rats in Germany, which might be explained by the wild-rat origin of the vast majority of rats in these breedings, which are mainly dedicated to the phenotypic testing of rodenticide resistance. The finding in this study that no SEOV was found in wild rats from Germany or the Netherlands together with previously reported SEOV strains from wild rats in Europe that were divergent from strains from captive rats [19,20] suggested that the transmission of SEOV is maintained within the captive rat population itself.

None of the wild and breeder rats from Germany tested positive for SEOV RNA or anti-SEOV IgG antibodies except for a single rat from North Rhine-Westphalia that tested positive in the hantavirus IgG-ELISA (C) and for PUUV in the line-blot assay, which suggested a PUUV spillover infection when taking into account that North Rhine-Westphalia is an endemic region for PUUV [49]. Similarly, six rats from the Netherlands tested positive in the hantavirus IgG ELISA (B), but none of them tested positive in the real-time RT-PCR or in the virus neutralization test (used on 4/6 rats). The discrepancy between the positive IgG ELISA results and the lacking reactivity in the neutralization assay might have been caused by the lower sensitivity of the latter, in particular when a hantavirus was not used in the assay that caused the infection, or might have been due to a false-positive result in the ELISA [28]. The failed detection of viral RNA in seropositive rats might have been caused by virus-clearance-mediated absence of viral RNA. A false-negative result in the real-time RT-PCR cannot be excluded for SEOV strains circulating in the Netherlands because few nucleotide exchanges in the viral RNA might cause problems in primer or probe binding.

The SEOV strain previously detected in wild rats in the Netherlands [20] differed from the strains found in the pet rats from Germany, the Netherlands, and France but showed more similarity to the wild-rat-associated Humber strain in Great Britain. This finding might suggest a common origin of SEOV strains in wild rats; however, it remains speculative at this point whether the strain(s) in wild rats have their origin in pet or breeder rats. On the other hand, a potential wildlife origin of pet-rat-associated strain(s) in Germany, France, the Netherlands, Great Britain, and the USA still remains to be determined. Such One Health investigations should focus on monitoring of rats as well as the molecular characterization of the source of human HFRS cases worldwide. This rat monitoring could profit from the workflow developed here but might apply highly sensitive RT-PCR assays targeting different SEOV strains.

## 5. Conclusions

The results of this study clearly indicated that pet rats might pose a risk for SEOV transmission in Europe. This risk of zoonotic transmission is even higher because the contact between pet rats and humans is closer than between wild or feeder/breeder rats and humans. Therefore, locating the source of the SEOV infection and monitoring pet rats for infection with SEOV is important to prevent the spread of the virus and zoonotic transmission to humans. In addition, patients with suspected (SEOV) hantavirus infections should be routinely questioned about their previous contact with rats (specifically pet rats).

## Figures and Tables

**Table 1:** Molecular and serological analysis of rats from patient/pet-rat owner 1 and from pet-rat owner 2.

Origin	ID	Sex	Conventional RT-PCR *			Serological Investigations			HTS	Accession Number
			SEOV S	SEOV M	SEOV L	IgG ELISA SEOV (A)	Hantavirus IgG ELISA (B)	Line-Blot **		
Pet-rat owner 1	KS19/1352	m	+	+	+	+	+	+	n.d.	OP870576 (S, par)
	KS19/1353	m	+	+	-	+	+	+	n.d.	n.a.
	KS19/1354 ***	m	+	+	+	+	+	+	spleen, liver	OP870570 (S, CDS); OP870572 (M, CDS); OP870574 (L, CDS)
	KS19/1414	f	-	n.d.	-	-	-	-	n.d.	n.a.
Pet-rat owner 2	KS19/2195	m	+	n.d.	n.d.	+	+	+	spleen, liver	OP870571 (S, CDS); OP870573 (M, CDS); OP870575 (L, CDS)
	KS19/2196	m	+	n.d.	n.d.	+	+	+	n.d.	n.a.
	KS19/2197	m	-	n.d.	n.d.	-	-	-	n.d.	n.a.

* Results of the conventional RT-PCR assays based on samples from lung, heart, liver, spleen, and kidney are given in Supplementary Table S2. ** Detailed line-blot reactivities of the samples are shown in Supplementary Figure S2. *** Initial detection of SEOV RNA from the first rat that caused the infection as previously described. Partial L and S segment sequences (accession numbers: MT123530 and MT123532, respectively) were already published [23]. Abbreviations: -, negative; +, positive; CDS, complete coding sequence; f, female; HTS, high-throughput sequencing; m, male; n.d., not done; par, partial sequence; n.a., not applicable.

**Table 2:** Results of serological and conventional RT-PCR analyses of different breeding colonies with wild rats or laboratory rats in Germany.

Rat Colony	Number	Species	Hantavirus IgG ELISA (B) **	Line-Blot Assay	SEOV-S RT-PCR
**A**	39	*R. norvegicus*	0/39	0/1	0/39
	33	*R. rattus*	0/33	0/1	0/33
**C**	24	*R. norvegicus*	0/24	0/3	0/24
	53	*R. rattus*	0/53	0/6	0/53
D *	22	*R. norvegicus*	0/22	n.d.	0/22
**E**	40	*R. norvegicus*	0/40	n.d.	0/40
**F**	277	*R. norvegicus*	0/276	n.d.	0/277
**G**	2	*R. norvegicus*	0/2	n.d.	0/2
**Total**	490		0/489	0/11	0/490

* Laboratory rats; ** used N proteins of DOBV, PUUV, HTNV, and SEOV; n.d., not done.

**Table 3:** Results of serological and conventional RT-PCR analyses of wild rats from different geographic regions in Germany.

Region *	Number	Species	Hantavirus IgG ELISA (B) **	Line-Blot Assay	SEOV-S RT-PCR
**North**	45	*R. norvegicus*	0/33	0/4	0/45
**West**	233	*R. norvegicus*	1 ***/220	1 ***/16	0/228
	1	*R. rattus*	0/1	n.d.	0/1
**South**	121	*R. norvegicus*	0/106	0/5	0/119
**East + Central**	163	*R. norvegicus*	0/150	0/9	0/152
**Total**	563		1/510	0/34	0/545

* North: Schleswig-Holstein and Hamburg; West: Lower Saxony, North Rhine-Westphalia, and Hesse; South: Baden-Wuerttemberg; East + Central: Mecklenburg-Western Pomerania, Brandenburg, Berlin, Saxony-Anhalt, and Saxony. ** Used N proteins of DOBV, PUUV, HTNV, and SEOV. *** Reactive in hantavirus IgG ELISA (B), but only reactive for PUUV in line-blot assay.

**Table 4:** Results of serological and real-time RT-PCR analyses of wildlife rats from different geographic regions in the Netherlands (Panel IV).

Study Location (s)	Year of Sample Collection	Location Type	Number	SEOV-IgG ELISA (C)	Real-Time RT-PCR
Amsterdam	2013–2014	Urban	57	2/57 *	0/2
Municipalities in the provinces of North-Brabant and Limburg	2016	Agricultural	47	0/47	n.d.
Appeltern, Roermond, and Maastricht	2018	Recreational water	80	4/80 **	0/80
Amsterdam	2020	Urban	136	0/136	0/136
Eindhoven	2020	Urban	5	0/5	0/5
Rotterdam	2021	Urban	269	n.d.	0/269
Total			594	6/325	0/492

* One black rat and one Norway rat were seropositive and subsequently analyzed using RT-PCR. ** All four seropositive rats tested negative in the virus neutralization assay using SEOV, DOBV, PUUV, and TULV. Note: n.d., not done.

## Data Availability

The relevant data are available upon reasonable request.

## Supplementary Materials

The following Supplementary Materials are available online at https://www.mdpi.com/1999-4915/15/2/467/s1, Table S1: Screening strategies of the different rat panels; Table S2: Tissue distribution of SEOV RNA determined via conventional RT-PCR in pet rats from pet-rat owner 1/patient and pet-rat owner 2; Table S3: Pairwise partial nucleotide (nt) and amino acid (aa) sequence similarities of the two novel SEOV strains to the SEOV reference strains; Table S4: Pairwise nucleotide (nt) and amino acid (aa) sequence similarities of the complete coding sequences and the entire proteins of the two novel SEOV strains to the SEOV reference strains; Figure S1: Results of the trace-back and trace-forward investigations of the origin of the SEOV infection in the pet rats; Figure S2: Results of the line-blot assay of the rat sera from the patient (pet-rat owner 1) and pet-rat owner 2; Figure S3: Phylogenetic tree of partial M segment sequences of the pet rats of the patient and the other owner deduced from the HTS dataset.
