# Supplementary material for: Pet Rats as the Likely Reservoir for Human Seoul Orthohantavirus Infection
Source: Viruses. 2023 Feb 7;15(2):467. doi: 10.3390/v15020467 (PMC9962845; doi:10.3390/v15020467)
Supplement: Supplementary file 1 [file viruses-15-00467-s001.zip › viruses-2145068-supplementary.pdf]

viruses MDPI

Supplementary Materials

Pet Rats as the Likely Reservoir for Human Seoul Orthohantavirus Infection

Table S1. Screening strategies of the different rat panels.

	Rat panel	Origin	Collection period	SEOV-IgG ELISA (A)*	Hantavirus IgG ELISA (B)**	SEOV-IgG ELISA (C)***	Line-blot assay	Conventional S RT-PCR	real-time RT-PCR
I	Pet rats	Germany	2019-2020	×	×		×	×[1]	
II	Breeder rats	Germany	2017-2020		×		×	×	
III	Wild-trapped rats	Germany	2007-2020		×		×	×	
IV	Wild-trapped rats	The Netherlands	2013-2021			×[2]			×[3]

* Based on Seoul orthohantavirus (SEOV) nucleocapsid protein [27]. ** Based on nucleocapsid proteins of Dobrava-Belgrade orthohantavirus (DOBV), Puumala orthohantavirus (PUUV), Hantaan orthohantavirus (HTNV) and SEOV. *** Based on commercial DOBV/HTNV IgG ELISA (Progen Biotechnik GmbH, Heidelberg, Germany), adapted by using rabbit-anti-rat horseradish peroxidase labeled IgG conjugate (Sigma–Aldrich Chemie B.V. Zwijndrecht, The Netherlands) at a 1:5000 dilution. [1] For pet rats from pet-rat owner 1/patient additionally M and L segment RT-PCR was performed. [2] Rats from 2021 were not tested with the ELISA, only by real-time RT-PCR. [3] Until 2018, real-time RT-PCR was only performed when rats were seropositive. Abbreviations: S, small; M, medium; L, large.

Table S2. Tissue distribution of Seoul orthohantavirus RNA determined by conventional RT-PCR in pet rats from pet-rat owner 1/ patient and pet-rat owner 2.

Tissue	Rats from pet-rat owner 1/patient												Rats from pet-rat owner 2								
	KS19/1352			KS19/1353			KS19/1354			KS19/1414			KS19/2195			KS19/2196			KS19/2197		
	S	M	L	S	M	L	S	M	L	S	M	L	S	M	L	S	M	L	S	M	L
lung	-	-	-	-	+	-	+*	+	+*	-	n.d.	-	+	n.d.	n.d.	-	n.d.	n.d.	-	n.d.	n.d.
heart	+	+	-	+	+	-	+	+	-	-	n.d.	-	+	n.d.	n.d.	+	n.d.	n.d.	-	n.d.	n.d.
liver	+	+	+	+	+	-	+	+	+	-	n.d.	-	+	n.d.	n.d.	-	n.d.	n.d.	-	n.d.	n.d.
spleen	+	+	+	+	+	-	+	+	+	-	n.d.	-	+	n.d.	n.d.	-	n.d.	n.d.	-	n.d.	n.d.
kidney	+	+	+	+	+	-	+	+	+	-	n.d.	-	+	n.d.	n.d.	+	n.d.	n.d.	-	n.d.	n.d.

* Previously investigated in [23]. Abbreviations: +, positive; **-**, negative; L, large segment; M, medium segment; n.d., not done; S, small segment.

Table S3. Pairwise partial nucleotide (nt) and amino acid (aa) sequence similarities of the two novel Seoul orthohantavirus (SEOV) strains to SEOV reference strains.

Origin	partial	S segment / N protein		M segment / GPC		L segment / RdRP	
	Reference sequence KS19/1354 (PRO-1/P)	nt (513)	aa (171)	nt (261)	aa (87)	nt (291)	aa (97)
GER	**KS19/2195 (PRO-2)**	1.0	1.0	1.0	1.0	1.0	1.0
USA	**MK360777, MK360793, MK360803**	1.0	1.0	1.0	1.0	0.996	1.0
GBR	**MZ343377, MZ343376, MZ343375**	0.998	0.994	1.0	1.0	0.996	1.0
FRA	**KX064270, KX064268**	1.0	1.0	n.a.	n.a.	0.996	1.0
NL	**MG764078, MG764080, MT993925**	1.0	1.0	1.0	1.0	0.993	1.0

Abbreviations: FRA, France; GBR, Great Britain; GER, Germany; GPC, glycoprotein precursor; L, large segment; M, medium segment; N, nucleocapsid; n.a. = not available; NL, the Netherlands; PRO-1/P, pet-rat owner 1/patient; PRO-2, pet-rat owner 2; RdRP, RNA-directed RNA polymerase; S, small segment.

Table S4. Pairwise nucleotide (nt) and amino acid (aa) sequence similarities of the complete coding sequences and the entire proteins of the two novel Seoul orthohantavirus (SEOV) strains to SEOV reference strains.

Origin	CDS	S segment / N protein		M segment / GPC		L segment / RdRP	
	**Reference sequence KS19/1354 (PRO-1/P)**	nt (1290)*	aa (429)	nt (3402)*	aa (1133)	nt (6456)*	aa (2151)
GER	**KS19/2195 (PRO-2)**	0.999	1.0	0.998	0.998	0.998	0.999
USA	**MK360784, MK360787, MK360806**	0.999	1.0	0.998	1.0	0.997	1.0
GBR	**MZ343377, KM948593, KM948594**	0.997	0.997	0.996	0.997	0.996	0.999
FRA	**KX064271**	1.0	1.0	n.a.	n.a.	n.a.	n.a.
NL	**MG764078, MG764080, MG764083**	1.0	1.0	0.998	1.0	0.989	0.991

Abbreviations: CDS, complete coding sequence; FRA, France; GBR, Great Britain; GER, Germany; GPC, glycoprotein precursor; L, large segment; M, medium segment; N, nucleocapsid; n.a. = not available; NL, the Netherlands; PRO-1/P, pet-rat owner 1/patient; PRO-2, pet-rat owner 2; RdRP, RNA-directed RNA polymerase; S, small segment. * including stop codon.

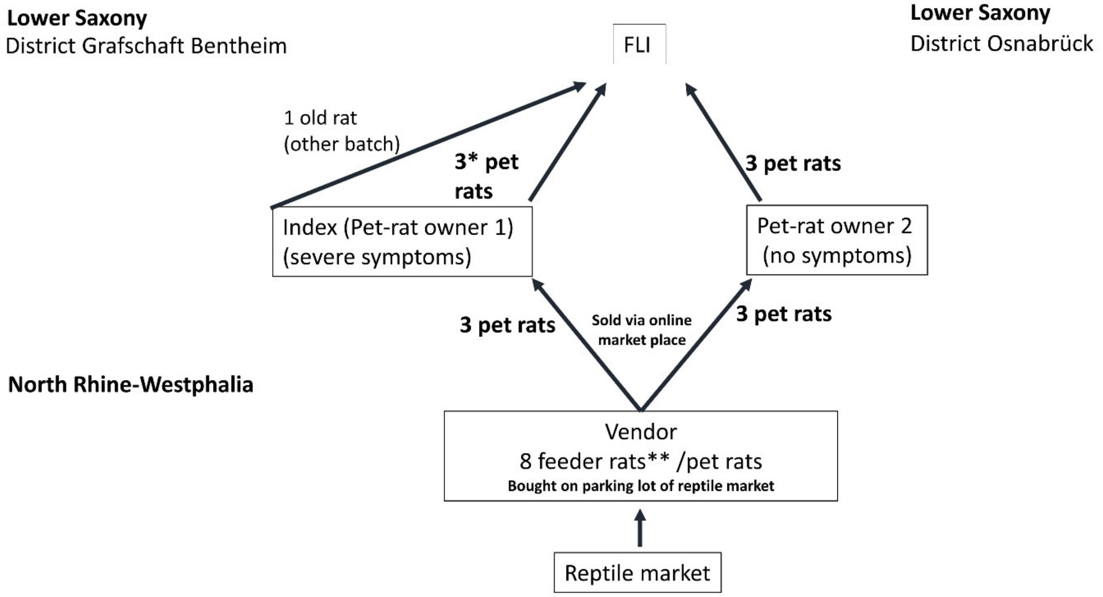

Figure S1. Results of the trace-back and trace-forward investigations of the origin of the Seoul orthohantavirus (SEOV) infection in pet rats. *Molecular SEOV detection in one rat was already published [23]. **Two feeder rats not sold, died. FLI, Friedrich-Loeffler-Institut.

Origin	ID
pet-rat owner 1/ patient (PRO-1/P)	KS19/ 1352
	KS19/ 1353
	KS19/ 1354*
	KS19/ 1414
pet-rat owner 2 (PRO-2)	KS19/ 2195
	KS19/ 2196
	KS19/ 2197
Immunized rats	Positive control L
	Positive control R
	Negative control

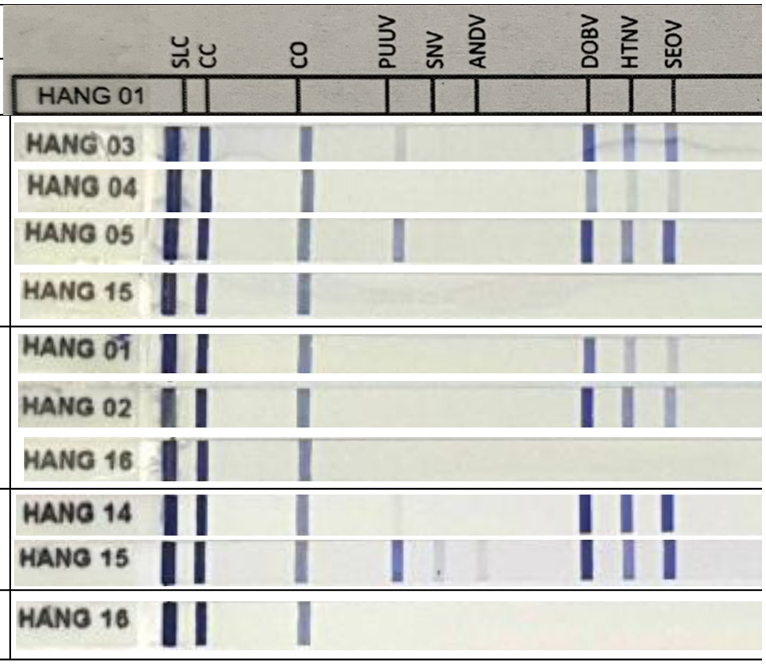

Figure S2. Results of the line-blot assay of the rat sera from the patient (pet-rat owner 1, PRO-1/P) and the pet-rat owner 2 (PRO-2). *Pet rat was previously tested with RT-PCR [23].

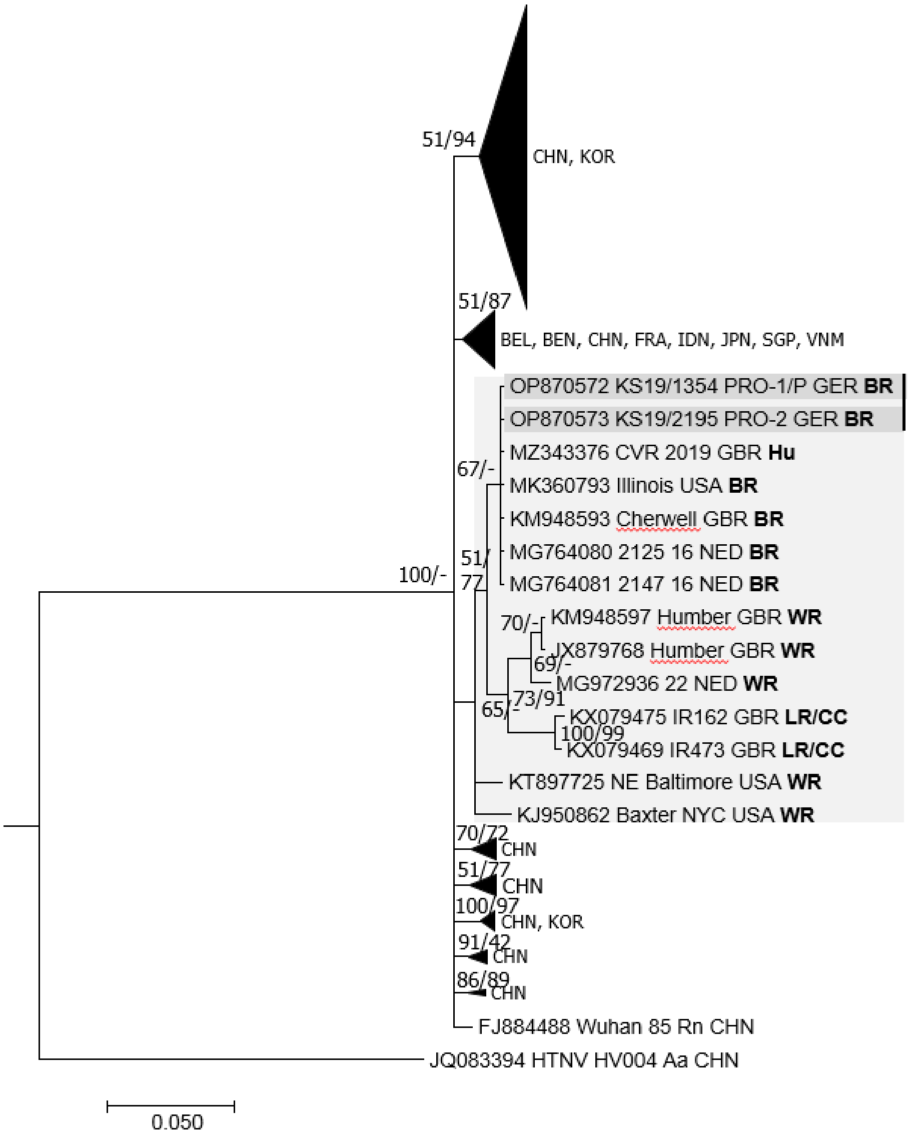

Figure S3. Phylogenetic tree of partial Seoul virus (SEOV) M segment sequences of the pet rats of the patient and the other pet rat owner, deduced from the high-throughput sequencing (HTS) dataset. The partial M segment consensus tree was based on Bayesian and maximum likelihood analyses with the GTR substitution model including gamma distribution, invariant sites, 8×10^6 generations and 1000 bootstrap replicates with an alignment length of 263 nucleotides. The consensus tree is based on the Bayesian method. Bootstrap values from the maximum likelihood analyses were only transferred to the consensus tree if the branches were identical. Posterior probabilities are given before and bootstrap values behind or below slashes. The relevant clade is highlighted by a light grey background. Sequences derived from the same outbreak are marked by a black bar, while novel sequences from the pet rats are indicated in darker grey. Country abbreviations: BEL, Belgium; BEN, Benin; CHN, China; FRA, France; GBR, Great Britain; GER, Germany; IDN, Indonesia; JPN, Japan; KOR, Korea; NED, the Netherlands; SGP, Singapore; USA, United States of America; VNM, Vietnam. Other abbreviations: HU, human; PRO-1/P, pet-rat owner 1/patient; PRO-2, pet-rat owner 2; Rn, *Rattus norvegicus*; BR, breeder rat; CC, cell culture isolate; LR, laboratory rat; WR, wild rat. Categorization of the rats according to [10,19,20,43–47].
